# Supplementary material for: A high-resolution mRNA expression time course of embryonic development in zebrafish
Source: eLife. 2017 Nov 16;6:e30860. doi: 10.7554/eLife.30860 (PMC5690287; doi:10.7554/eLife.30860)
Supplement: Supplementary file 6. [file elife-30860-supp6.zip › biolayout-clusters-files/Cluster103.html]

Cluster103


# Cluster103: Detail

### Go to ZFA detail

## GO

| GO ID | Description | Domain | Annotated | Expected | Observed | Adjusted p-value | Genes | Ensembl IDs |
| --- | --- | --- | --- | --- | --- | --- | --- | --- |
| GO:0004867 | serine-type endopeptidase inhibitor acti... | molecular\_function | 40 | 0.05 | 4 | 4.1e-05 | serpinc1 itih2 serpina1 serpina1l | ENSDARG00000042684 ENSDARG00000045516 ENSDARG00000090286 ENSDARG00000090850 |

  


### Go to GO detail

## ZFA

| ZFA ID | Description | Annotated | Expected | Observed | Fold Enrichment | Adjusted p-value | Genes | Ensembl IDs |
| --- | --- | --- | --- | --- | --- | --- | --- | --- |
| ZFA:0000088 | YSL | 581 | 0.61 | 7 | 11 | 0.0067 | serpinc1 adh8b itih2 dao.2 serpina1l MPC2 (1 of many) oxct1b | ENSDARG00000042684 ENSDARG00000024278 ENSDARG00000045516 ENSDARG00000035603 ENSDARG00000090850 ENSDARG00000037679 ENSDARG00000054319 |
